# Realising the potential of the family history in risk assessment and primary prevention of coronary heart disease in primary care: ADDFAM study protocol

**DOI:** 10.1186/1472-6963-9-184

**Published:** 2009-10-12

**Authors:** Nadeem Qureshi, Sarah Armstrong, Paula Saukko, Tracey Sach, Jo Middlemass, Phil H Evans, Joe Kai, Hannah Farrimond, Steve E Humphries

**Affiliations:** 1 Division of Primary Care, University of Nottingham, Nottingham, UK; 2 Trent Research and Development Support Unit, University of Nottingham, Nottingham, UK; 3 Department of Social Sciences, Loughborough University, Loughborough, UK; 4 School of Chemical Sciences and Pharmacy, University of East Anglia, Norwich, UK; 5 Research and Development Department, Nottinghamshire County Teaching Primary Care Trust, Nottingham, UK; 6 Peninsula Medical School, Universities of Exeter and Plymouth, UK; 7 EGENIS (ESRC Centre for Genomics in Society), University of Exeter, Exeter, UK; 8 Centre for Cardiovascular Genetics, British Heart Foundation Laboratories, Royal Free and University College London Medical School, London, UK

## Abstract

**Background:**

Coronary heart disease (CHD) is the leading cause of death in the developed world, and its prevention a core activity in current UK general practice. Currently, family history is not systematically integrated into cardiovascular risk assessment in the UK, Europe or the US. Further, primary health care professionals' lack the confidence to interpret family history information and there is a low level of recording of family history information in General Practice (GP) records. Primary prevention of CHD through lifestyle advice has sometimes yielded modest results although, for example, behavioural interventions targeted at "at risk" patients have produced encouraging findings. A family history approach, targeted at those requesting CHD assessment, could motivate lifestyle change. The project will assess the clinical value of incorporating systematic family history information into CHD risk assessment in primary care, from the perspective of the users of this service, the health care practitioners providing this service, and the National Health Service.

**Methods/Design:**

The study will include three distinct phases: (1) cross-sectional survey to ascertain baseline information on current recording of family information; (2) through an exploratory matched-pair cluster randomised study, with nested qualitative semi-structured interview and focus group study, to assess the impact of systematic family history recording on participants' and primary care professionals' experience; (3) develop an economic model of the costs and benefits of incorporating family history into CHD risk assessment.

**Discussion:**

On completion of the project, users and primary care practitioners will be more informed of the value and utility of including family history in CHD risk assessment. Further, this approach will also act as a model of how familial risk information can be integrated within mainstream primary care preventive services for common chronic diseases.

**Trial Registration:**

Current Controlled Trials ISRCTN17943542

## Background

Coronary heart disease (CHD) is the leading cause of death in the developed world, and its prevention a core activity in current UK general practice[1]. Family history is a significant risk factor for CHD, with, for example, 30% of UK middle-aged men reporting a family history of CHD experiencing a 71% higher CHD rate over the next 10 years, which remained unchanged after adjusting for classical risk factors such as cigarette smoking, hypertension, and obesity[2]. Similar findings have been reported in the US [3]. European prevention of CHD guidelines recommend that family history should be used as a primary prevention approach for CHD[4], while the CDC in the US has identified CHD as one of five conditions where identification of familial risk and appropriate intervention could significantly improve health[5]. This initiative has led to colleagues at the CDC commissioning a multi-centre clustered randomised controlled trial to evaluate the utility of a robustly developed CDC family history tool and intervention software ("Family HealthWare") with US Primary Care Providers[6]. The tool appears promising but the trial will not assess the utility of incorporating family history into standard primary prevention risk assessment tools.

Currently, family history is not systematically integrated into CHD risk assessment in the UK, Europe or the US[1,7]. In the UK, patients who could benefit from primary prevention of CHD are usually identified using standard CHD risk assessment scores that incorporate age, gender, diabetes, smoking status and blood pressure levels[7]. Specifically family history collection should identify relatives with premature history of CHD. The Joint British Societies guidelines note that "a family history of premature CHD (male first degree relatives aged under 55; female first degree relatives aged under 65) increases the patient's risk by a factor of approximately 1.5" but this is not explicitly incorporated in the CHD risk prediction charts[1]. Our previous research has found that primary care professionals' lack the confidence to interpret family history information[8,9] and there is a low level of recording of family history information in General Practice (GP) records[10].

Primary prevention of CHD through lifestyle advice has sometimes yielded modest results[11] although, for example, behavioural interventions targeted at "at risk" patients have produced encouraging findings[12]. There is evidence that interventions that have targeted the family as a whole, rather than individuals, are more effective[13], and it has been reported that young individuals who were aware of their family history of CHD were less likely to smoke[14]. This evidence suggests that a family history approach, targeted at "at risk" individuals and those requesting CHD assessment, could motivate lifestyle change. However, it has been suggested that assessment of inherited risk may produce fatalism or anxiety[15], although these findings have not been corroborated in other studies[16]. Furthermore, people from different backgrounds have different understanding of the causes of heart disease; research has also discovered varied "lay models" of the risk associated with a family history of heart disease [17-19]. In light of these findings, it has been emphasized that to be effective, CHD prevention needs in-depth sensitivity to the ethnically and socioeconomically diverse patients' personal and cultural models of disease and family history [17,20]. Still, patients' interpretations of, and reactions to, incorporating family history into CHD risk assessment have not been studied. We have previously explored the psychological impact of a general family history questionnaire (FHQ)[21] and the validation of this instrument [22]. We have completed a qualitative pilot study on the feasibility of incorporating family history into CHD assessment from a risk communication point of view [23]. However, in order to inform policy the assessment of the clinical utility of using family history is now needed as a priority, alongside work such as that by the CDC Family HealthWare project, and prior to the development *de novo *of further tools[6]. This should include examination of the behavioural and psychological effects of CHD interventions using family history assessment. Moreover a better understanding of the implications of being identified as at higher risk of heart disease (due to a family history of premature CHD), for individuals from different social and ethnic backgrounds is also needed.

The project will assess the clinical value and utility of incorporating systematised family history information into Coronary Heart Disease (CHD) risk assessment in primary care, from the perspective of the users of this service, the health care practitioners providing this service, and the National Health Service. Current CHD risk assessment guidelines express CHD risk within cardiovascular disease (CVD) risk assessment, with patients divided into three broad risk categories based on the risk of CVD in the next 10 years: Average risk (less than 10% CVD risk), Moderate risk (10% to 19% CVD risk), and High risk (20% or greater 10 year CVD risk)[1].

This three-phase project will assess the clinical value of incorporating systematic family history information into the standard CVD risk assessment in General Practice, by:

1 Estimating the extra proportion of participants at high risk of CHD, who would benefit from intensive lifestyle advice and medications, when incorporating systematically collected family history into the risk assessment, in comparison with current practice.

2 Comparing changes in self-reported behaviour, anxiety and social/psychological experience, between individuals who have been allocated to either CVD risk assessment using (i) standard risk assessment complemented by systematic collection of family history or (ii) standard risk assessment alone (usual care).

Further, there will be an assessment of general practitioners' and practice nurses' views on the feasibility and acceptability of incorporating family history into routine CVD risk assessment.

To achieve these goals, the project will include three distinct phases: (1) ascertain baseline information on current recording of family information, (2) assess, through an exploratory randomised study (including quantitative measures, qualitative semi-structured interviews and focus groups), the impact of systematic family history recording on participants' and primary care professionals' experience, and (3) develop an economic model of the costs and benefits of incorporating family history into CVD risk assessment. Phase 1 will provide information on the impact of CHD family history currently available in GP records on CVD risk assessment, with which the systematic collection of CHD family history (in Phase 2) will be compared, to assess the additional value of the latter intervention. Phase 3 will assess the benefits of systematic collection through health economic analyses.

Through the above three phases it is anticipated that the following five outcomes will be evaluated:

### Outcome (1)

An estimate of the proportion of the population who are at a 20% or greater CVD risk in the next 10 years with and without inclusion of CHD family histories currently collected in GP records. [Phase 1]

### Outcome (2)

An estimate of the proportion of the population who are at a 20% or greater CVD risk in the next 10 years, comparing current practice to systematic collection of family history in the intervention practices. [Phase 2]

### Outcome (3)

A comparison of self-reported behaviour (smoking, exercise, diet), psychological impact (anxiety, fatalism), perception of health, psychological and social experience between participants who have undergone CVD risk assessment using (i) standard risk assessment alone or (ii) standard risk assessment complemented with systematic collection of family history. [Phase 2]

### Outcome (4)

An evaluation of the acceptability and feasibility to general practitioners and practice nurses of incorporating formal family history collection into their routine CVD assessment. [Phase 2]

### Outcome (5)

A cost-effectiveness analysis of systematic family history collection compared to current practice will be undertaken from an NHS primary care perspective over the trial period. Parameters for the analysis will come from phase 1 and 2 of the project, supplemented with data from the published literature where necessary. [Phase 3]

On completion of the project, users and primary care practitioners will be more informed of the value and utility of including family history in CVD risk assessment. Further this approach will also act as a model of how familial risk information can be integrated within mainstream primary care preventive services for common chronic diseases.

## Method/design and discussion

To achieve the five research outcomes the project will proceed through three distinct phases. All phases of the study received ethical approval from the Multi Centre Research Ethics Committee for Scotland.

## PHASE 1: BASELINE CVD RISK ASSESSMENT WITH/WITHOUT CURRENTLY AVAILABLE INFORMATION ON CHD FAMILY HISTORY IN GP RECORDS

During this phase, the inclusion of currently collected information about family history of premature CHD in General Practice records on CVD risk assessment will be assessed. The results of this phase will inform outcome 1. This entails a cross-sectional survey to examine the current collection and retrieval of family history data in GP manual records. The impact on absolute CVD risk scores of premature CHD family history will be informed by consensus. Using GP records, standard CVD risk assessment will be compared with risk assessment resulting from the inclusion of previously collected CHD family history in GP records.

### Methods

#### Consensus agreement

absolute risk of CHD associated with premature CHD in first and second degree relatives will be identified through expert panel and literature review using Joint British Societies' guidelines as a baseline[1]. As well as the project team, the panel will include the following experts: Cardiovascular epidemiologists [John Yarnell, Queens University Belfast (who has been involved in several prospective CHD studies and on MONICA projects for CHD), Public health genetic epidemiologist (Paula Yoon, CDC), General Practitioner with a track record of assessing CHD genetic morbidity in Primary Care (Dr Ian Hopkinson), Public health academic with special interest in Genetics (Brenda Wilson, Ottawa University) and Community geneticist (Judith Allanson, Ottawa University)].

#### Outcome measures data collection

Current levels of recording of premature CHD family history in GP computer records for participants recruited in Phase 2; supplemented by data from research team's previous studies, and literature review. The calculation of CVS risk will follow current JBS 2 guidelines[1].

#### Outcome measures data interpretation

Calculate current level of recording of relevant CHD family histories in GP computer records of participants entered in phase 2; Establish baseline distribution of recruited participants with average, moderate or high CVD risk categories, (less than 10%; 10 to 19%; 20% or greater CVD risk in next 10 years) on standard CVD risk assessment[1]. Recalculate the proportion at 20% or greater CVD risk after the inclusion of family history data routinely collected in GP records.

#### Sample Size

The proportion of the population at high CVD risk (20% and greater CVD risk over the next 10 years) has been estimated to be at least 5%[24]. From our previous studies in which GP records were reviewed, between 3% and 13% fulfil Joint Society family history guidelines[1]. Based on this data it is estimated that the proportion of population at high CVD risk, after the inclusion of previously collected family history in the calculation, will increase by 1% to 6% (in the age group 30 to 65). To estimate an expected percentage of 6% with a two-sided 95% confidence interval extending from 3.5 to 8.5% a total of 347 participant GP manual records need to be reviewed. The small anticipated increase acknowledges the poor recording of this information, and the assumption that not all participants who are actually at higher familial risk will shift into the high CVD risk category.

## PHASE 2: IMPACT AND ACCEPTABILITY OF SYSTEMATIC FAMILY HISTORY COLLECTION: AN EXPLORATORY MATCHED-PAIR CLUSTER RANDOMISED STUDY

This phase of the project seeks to evaluate the impact of systematically collecting relevant premature CHD family histories. The data collected will be both quantitative, in terms of the number of extra participants identified and self-reported behavioural and psychological measures, and qualitative, in terms of the experience of participants and professionals involved. The results of this phase will inform outcomes 2 and 5 and achieve outcomes 3 and 4. Qualitative interviews will explore the processes that lead to the quantitative outcomes and evaluate social, cultural, service-related factors and ethical concerns that affect the acceptability and effectiveness of the intervention. Qualitative research has successfully been incorporated into randomised studies testing complex behavioural interventions in order to explore the acceptability of the intervention[25] and to examine the psychosocial processes that lead to the quantitative outcomes[26,27]. Focus groups will facilitate a participatory dialogue between clinicians on the acceptability and feasibility of the intervention[28].

### Methods

#### Study Design

**Figure 1:**
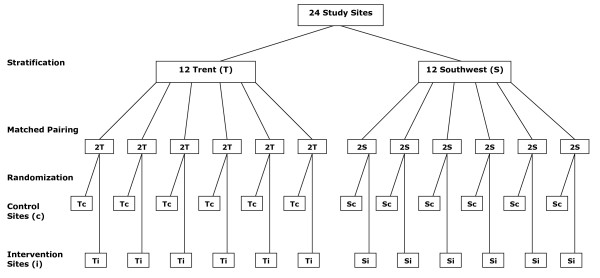
**Matched-Pair Allocation Scheme**. T = Trent, S = Southwest, Tc = control site in Trent, Sc = control site in Southwest, Ti = intervention site in Trent, Si = intervention site in Southwest.

A pragmatic exploratory matched-pair cluster randomised study with a nested qualitative semi-structured interview and focus group project. Practices from the Trent and Peninsula research networks will be invited to participate in the study. Researchers at both sites will visit interested practices to explain the research project. Twelve pairs of eligible practices, who agree to participate, will be recruited (6 pairs from South West England and 6 pairs in the East Midlands). The practices will be paired according to level of deprivation (as per Indices of Multiple Deprivation (IMD) quintiles), and ethnicity (as per more or less than 10% non-White population) in their specific electoral wards. One of each pair will be randomly assigned to the standard CVD risk assessment procedure, whilst the other practice within the pair, in addition to providing a standard CVD risk assessment, will also take account of premature CHD familial risk using the family history questionnaire (FHQ). Randomisation will be stratified by study centre. The matched-paired allocation scheme is demonstrated in figure 1. The randomisation list will be prepared centrally by an independent data manager at the University of Nottingham Clinical Trials Support Unit (CTSU). The random allocation sequence will be generated by a computer programme and the treatment allocation for each practice will be obtained by a password-protected internet link. The practices, research staff and participants will not be blinded to group assignment but the study statistician will be. For the statistical analysis treatment arms will be distinguished by a code assigned by the independent data manager at the CTSU. The meaning of these codes will not be revealed to any members of the research team until data analysis is complete. Figure 1 indicates the matched-pair allocation scheme used in this study.

The nested qualitative study will recruit participants in both the intervention and control arms, who have been assessed to be at high risk of heart disease (20% or above risk in the next ten years). We expect the impact of the assessment to be strongest in this group, and qualitative interviews will yield insights into how participants cope with being defined as at high risk, what behavioural and psychological impact this risk information has and how the response is mediated by individual and social contexts. Conducting the interviews with participants in both intervention and control arms allows for exploring whether family history assessment renders the participants' experience different and how.

After the recruitment for the study has been completed, six practices in the intervention arm (3 in the East Midlands and 3 in the South West) will be invited for a focus group. The focus groups will seek to solicit clinicians' views on the acceptability, feasibility and usefulness of the family history assessment; it will also invite the clinicians to discuss their usual practice in terms of taking family history of heart disease.

#### Study interventions

##### FHQ group

Detailed CHD family history information will be collected by participants completing a CHD focused validated Family History Questionnaire (FHQ) (already developed and evaluated by the research team)[22]. The effect of incorporating this information into the standard CVD risk assessment procedure and the subsequent advice given to participants on CVD risk and lifestyle will be assessed.

##### Control group

Participants will have their CVD risk assessment score calculated using the standard CVD risk assessment procedure.

##### Details of planned interventions

Participating clinicians in both groups will be given standardised training, including interpreting CVD risk, standard evidence/consensus based messages for lifestyle change. Clinicians in the FHx will in addition, be given information on interpreting and communication about the risk associated with family history of premature CHD, based on our pilot study[23]. To enhance the standardisation of the training, initial training sessions in both arms on both study sites will be video recorded, compared and fed back to the trainers.

#### Eligibility criteria

The eligibility criteria will ensure the inclusion of multi-ethnic and socio-economically deprived areas.

#### Practice level eligibility criteria

##### Inclusion Criteria

▪ To be within either Trent Primary Care Trusts in Nottinghamshire and Lincolnshire or the area served by the Peninsula Primary Care Research Network in Devon and Cornwall.

##### Exclusion Criteria

▪ General Practices outside of these PCTs/networks will be excluded.

#### Participant level eligibility criteria

##### Inclusion criteria

▪ Patients between 30 and 65 years of age.

##### Exclusion criteria

▪ Patients noted to have a previous history of atherosclerotic disease (this includes CHD, CVA & Peripheral Vascular Disease)

▪ Previous history of Diabetes Mellitus

▪ Patients already on statin therapy or other lipid lowering medications

▪ Patients considered by the General Practitioners to be inappropriate to recruit due to psychosocial and other reasons.

#### Recruitment

Patients, who are offered CVD risk assessment and referred for a cholesterol test as part of their normal care (either at their doctor's initiative or at their own request), will be invited to participate. These patients will be recruited opportunistically, as they present to the GP and practice nurse for a CVD risk assessment. They will be given a study pack, which includes consent forms, participant information sheet, collection of information on CHD risk factors (age, gender, ethnicity) and baseline outcome measures. Participants in the FHQ group will be given, in addition, the Family History Questionnaire. The completed baseline questionnaires and consent forms will be sent to the research team or handed to the practice receptionist, who will forward them to the research team.

#### Data collection procedures

After initial recruitment, all participants will have their medical records reviewed to reconfirm their eligibility. On arrival of the cholesterol blood results, CVD risk scores will be calculated by the research team, as per JBS2 guidelines[1]. In the FHQ group, familial CHD risk will also be calculated and incorporated into the overall score. Two weeks after the original consultation both clinicians and participants will be sent a letter informing them about the participant's risk status. Participants identified at average or moderate CVD risk in both groups will be sent a letter confirming status together with a leaflet on lifestyle advice. In addition to a leaflet, those at higher risk in both groups will be informed that they are at higher than average CVD risk and invited for a consultation in the practice within the next 2 weeks.

In the consultation with doctor or nurse, participants will have their CVD risk explained and given lifestyle advice and, when clinically indicated, statins will be offered, as per usual practice. Participants in the FHQ group will also have the impact of premature CHD family history on the CVD risk score explained.

At two weeks and six months after the second consultation (for high risk participants) or letter being sent (for average or moderate risk participants) all participants will be posted a set of questionnaires. Participants, who have been identified at high risk, will be invited for a qualitative interview by phone, following a purposeful, maximum variation sampling strategy in terms of being in intervention or control arm, gender, age, occupational class, ethnicity, region and having or not having a family history of premature CHD. Recruitment will continue until thematic saturation is reached. Participants in the qualitative study will be interviewed by an experienced researcher in their homes (or if the participants so desire by phone) at 2 weeks and 6 months after the consultation in the practice.

Six practices in the intervention arm will be invited to participate in a focus group to capture the practitioners' views on using family history assessment and incorporate this into their clinical decision making.

After nine months the recruited participants' computer GP records will be reviewed for prescribing of: lipid lowering, anti-obesity, and anti-thrombolytic drugs, as well as nicotine replacement therapy.

#### Outcome measures

The primary outcome measure is the proportion of participants falling in the high risk of CVD category

The secondary outcome measures are:

i) Self-reported lifestyle changes; including changes in smoking habits, levels of exercise and fat intake[29,30]

ii): Anxiety, using the 6 item Spielberger State-Trait Anxiety Inventory (STAI)[31] and fatalism and causal attributions of heart disease using the Illness Perception Questionnaire[32,33]

iii): Perception of health-related quality of life (SF-6D, derived from SF-36, and global question 2)[34,35].

The first two groups of these measures have been used in the assessment of CHD prevention in UK primary care. Perception of health and 6 item STAI measures have been used in other studies on family history and genetic screening in primary care[21,36,37].

Qualitative interviews will be used to analyse the contextual processes that mediate lifestyle change and risk perceptions of participants identified as at high risk in both the control and the FHQ group of the study. Participants' changes in views and their perceptions of heart disease, family history, their personal risk, lifestyle, medications, ethical concerns and their social, cultural and familial context will be collected.

#### Data management

The FHQs and outcome questionnaires will be anonymised, given a unique identifier and then entered onto a database. An Access database will be specifically designed to monitor returns from each participating practice using consecutive numbering. The identifiable data will be stored securely at one site and this information will not be linked to actual socio-economic data, CVD risk assessment variables, family history and outcome measures; the unique identifier will be used for this purpose.

The Access administration database will include fields for age, gender, consent to participate, post code and sub-sample agreeing to be interviewed. Each week a printout will be generated providing details of participants who need to be contacted and sent the postal survey. The returning data will then be entered onto the main database using the participant's unique identification number.

Quality control in data entry: a 1 in 2 sample of the first 200 lifestyle outcome questionnaires at baseline and 2 weeks will be rechecked to identify errors in entry. Error rates greater than 0.5% will lead to double checking of all data entry. All CVD risk assessment scores will be checked by a second member of the team.

Interview and focus group audiotapes will be transcribed verbatim (no names or other identifying details will be included), and stored separately and securely away from transcripts.

The data will be stored for ten years on completion, in a locked filing cabinet within the researcher's employing organisational premises, in accordance with the Sponsor's requirements. Electronic data will be stored in an anonymised format and will also be encrypted.

All members of the research team will work within the principles of the Data Protection Act (1998) and Caldicott and Research Governance.

#### Sample size

The sample size for the primary outcome measure is based on the comparison of the change in the percentage of participants classified as being at high CVD risk between treatment groups. The calculation assumes that the proportion of participants at high risk will increase by 3% in the FHQ group and that there will be no increase in the standard risk assessment (control) group. Assuming a power of 80% and a two-tailed alpha of 0.05, 265 participants per group are required to detect a difference of 3%. To allow for the cluster design, an ICC of 0.01 and a cluster size of 40 are assumed. This gives a sample size of 369 participants per group completing CVD risk assessment (i.e. 10 practices per group). This is a conservative estimate since the increase in precision due to the matching of practices is not allowed for as it was difficult to find an estimate of the ICC from matched-pair data.

Further, up to 40 individuals fulfilling the "high risk" criteria, either incorporating or not incorporating family history, will be invited for qualitative interviews (this will be sufficient to reach thematic saturation in major subgroups). Focus groups will be conducted with clinicians at six practices in the FHx arm (approximately 35 individuals in total), which is also estimated to be sufficient for thematic saturation.

#### Attrition rate

One thousand four hundred patients will be invited to participate. It is estimated that one thousand participants will complete consent and baseline questionnaire, with eight hundred completing the CVD risk assessment by having the serum cholesterol test, resulting in 400 participants recruited in each arm of the study.

Further, related to secondary outcome measures, it is estimated that 60% of recruited participants will complete the 6 month questionnaire. Hence 300 participants in each arm are expected to complete the study.

#### Data analysis

Quantitative data: Analysis will be undertaken on an intention to treat basis in that participants will be analysed in the groups to which they were allocated. The primary time point of interest is 6 months. Practices will be included in the analysis provided complete data (the outcome of interest at baseline and at 6 months follow up, and participant/practice level data that will be adjusted for in the analysis) are available for at least one of the participants recruited to the practice. Participants within the practice with incomplete data will be excluded from the analysis. If data for the secondary outcomes are missing for the 6 month assessment, a sensitivity analysis will be performed in which data from the two week assessment will be carried forward. An analysis assuming the worst case scenario of the 6 month assessment value being the same as the baseline value will also be performed.

The distribution of participants in average, moderate and high risk categories will be described by treatment group. The increase in the proportion of participants at high risk of CVD in the next 10 years as a result of incorporating systematically collected family history into CVD risk assessment score will be presented. For the primary outcome measure and the secondary outcome measures that are binary variables, the statistical significance of the difference in the event rates (e.g. the mean proportion of participants at high risk) between the FHQ and control groups will be calculated using a weighted paired t-test after the adjustment for the pairing of practices, participant and practice level variables that are associated with the outcome of interest. A weighted paired t-test will be used because it is anticipated that there will be wide variation in the number of participants recruited per practice. The robustness of the t-test will be assessed using a permutation test. Continuous outcome variables will be analysed using a similar method with differences in the mean response between the FHQ and control groups being compared using a weighted paired t-test. 95% confidence intervals will be presented for the intervention effect for all outcome measures.

Qualitative data: Interviews will be analysed using the constant comparative method[38]. Identifying emergent themes and developing the interview schedule and theory throughout the research process. It is foreseen that the qualitative project will identify psychosocial process as well as aspects of the intervention that mediate the salience of the lifestyle messages, but the open-ended nature of the interview process is designed to capture other relevant themes that emerge. The coding of the themes will be facilitated by the use of NVivo qualitative software, the thematic analysis during the research process and in the formal coding phase will be inter-rater validated between experienced qualitative researchers, supplemented by input from primary care practitioners. The analysis will also explore differences between participants from the two arms of the study, and with different socioeconomic and ethnic background. In the final stage, the qualitative findings will be triangulated with the quantitative data (including patient records) to facilitate a deeper interpretation of both data sets. Focus groups will also be analysed using the constant comparative method as described above but taking into account the conversational patterns occurring in the focus groups[28]. The topic guide of the discussion groups will focus on the feasibility and acceptability of the intervention for professionals but is open for other themes that may emerge.

#### Ethical considerations

We do not foresee major ethical issues arising from our study. Filling the FHQ may transiently raise patients' anxiety[21], but we are recruiting patients who have either requested CVD risk assessment themselves or been offered it by their doctor, so we are not increasing anxiety in a previously unaware population. We will follow normal ethical procedures to ensure informed consent, confidentiality and data protection.

## PHASE 3: THE FEASIBILITY OF INCORPORATING CHD FAMILY HISTORY INTO CHD RISK ASSESSMENT IN PRIMARY CARE: AN ECONOMIC PERSPECTIVE

### Overview

The resources available to provide health care are limited and therefore, it is important to establish if incorporating the systematic family history tool into CVD risk assessments is both effective and cost effective, that is, does the family history tool offer value for money. This phase will establish the costs of the intervention and the cost consequences of using the family history tool alongside estimating the benefits of using the tool. Using data from phases 1 and 2, (and if necessary other published data), an economic evaluation of the systematic incorporation of family history into CVD risk assessment compared to current practice from an NHS primary care perspective will be undertaken over the trial period. Established and accepted economic methodologies will be employed throughout[39]. This phase will achieve outcome 5 of the study.

### Methods

A cost analysis will be undertaken. Resource items likely to change as a result of the intervention (as well as the cost of developing and providing the intervention) will be identified and measured (collected using MIQUEST software) during phase 1 and 2. Unit costs will be derived from national published data[40,41] and used to value the resources measured for a common price year.

If the family history tool is to improve outcomes it not only needs to identify more at-risk individuals at an earlier stage (although this is likely to induce some benefit compared to usual practice in and of itself) but also lead to behavioural change. Such behavioural change is likely to take time as people go through stages of change towards permanent change. Therefore, the six month period of the trial may only be sufficient to detect the change in numbers identified rather than any actual change to health-related quality of life brought about through behavioural change. To test if this is in fact the case, two outcomes will be measured and two approaches to economic evaluation employed. This study will estimate the cost-effectiveness in terms of cost per additional high risk individual identified and the cost utility using the SF-6D administered at baseline and 6 months[35] to estimate the cost per QALY over the trial period (with use of 2 week score for sensitivity analysis, if 6 month score not available). Both analyses enable technical efficiency questions to be addressed, that is how best to identify those at high risk of CHD (applying a systematic approach to family history collection or not) whilst the cost-utility analysis will also enable allocative efficiency questions to be addressed, that is whether resources spent on using a systematic approach to CHD family history collection are considered cost-effective compared to a diverse range of other health interventions or services.

The timeframe for the economic analysis will be that of the trial period. If non-dominance occurs (that is if costs are greater and the intervention is more effective or if the intervention is cheaper and less effective) an incremental cost-effectiveness ratio (the ratio of change in cost divided by change in benefit comparing the systematic approach to family history collection versus usual care) will be produced. The confidence region around the incremental cost effectiveness ratio will be estimated and presented using appropriate statistical techniques[42]. Appropriate sensitivity analyses will be undertaken to test the robustness of the results.

## Abbreviations

CDC: Centre for Disease Control and Prevention; CHD: Coronary Heart Disease; CVA: Cerebral Vascular Accident; CVD: Cardiovascular Disease; FHQ: Family History Questionnaire; FHx: Family History; ISCTRN: International Standardised Randomised Controlled trials Number; NICE: National Institute for Health and Clinical Excellence; PCTs: Primary Care Trusts.

## Competing interests

The authors declare that they have no competing interests.

## Authors' contributions

The study was conceived through discussions between NQ, PS and SH. Original study protocol was drawn up by NQ, SA and PS. The manuscript was further modified by JM, TS, JK, PE and HF. All authors read and approved the final manuscript.

## Pre-publication history

The pre-publication history for this paper can be accessed here:


